# Sex and Exposure to Postnatal Chlorpyrifos Influence the Epigenetics of Feeding-Related Genes in a Transgenic *APOE* Mouse Model: Long-Term Implications on Body Weight after a High-Fat Diet

**DOI:** 10.3390/ijerph18010184

**Published:** 2020-12-29

**Authors:** Laia Guardia-Escote, Jordi Blanco, Pia Basaure, Judit Biosca-Brull, Rikst Nynke Verkaik-Schakel, Maria Cabré, Fiona Peris-Sampedro, Cristian Pérez-Fernández, Fernando Sánchez-Santed, Torsten Plösch, José L. Domingo, Maria Teresa Colomina

**Affiliations:** 1 Research in Neurobehavior and Health (NEUROLAB), Universitat Rovira i Virgili, 43007 Tarragona, Spain; laia.guardia@urv.cat (L.G.-E.); jordi.blanco@urv.cat (J.B.); piaisabel.basaure@alumni.urv.cat (P.B.); judit.biosca@urv.cat (J.B.-B.); maria.cabre@urv.cat (M.C.); 2 Department of Psychology and Research Center for Behavior Assessment (CRAMC), Universitat Rovira i Virgili, Campus Sescelades, 43007 Tarragona, Spain; 3 Laboratory of Toxicology and Environmental Health, School of Medicine, IISPV, Universitat Rovira i Virgili, 43201 Reus, Spain; joseluis.domingo@urv.cat; 4 Department of Basic Medical Sciences, Universitat Rovira i Virgili, 43201 Reus, Spain; 5 Department of Obstetrics and Gynecology, University Medical Center Groningen, University of Groningen, 9713 GZ Groningen, The Netherlands; r.n.schakel@umcg.nl (R.N.V.-S.); t.plosch@umcg.nl (T.P.); 6 Department of Biochemistry and Biotechnology, Universitat Rovira i Virgili, 43007 Tarragona, Spain; 7 Department of Physiology/Endocrinology, Institute of Neuroscience and Physiology, The Sahlgrenska Academy at the University of Gothenburg, 405 30 Gothenburg, Sweden; fiona.peris.sampedro@gu.se; 8 Department of Psychology and Health Research Center (CEINSA), Almeria University-ceiA3, 04120 Almeria, Spain; cpf603@ual.es (C.P.-F.); fsanchez@ual.es (F.S.-S.)

**Keywords:** chlorpyrifos, *APOE*, epigenetics, feeding control, high-fat diet

## Abstract

Developmental exposure to toxicants and diet can interact with an individual’s genetics and produce long-lasting metabolic adaptations. The different isoforms of the apolipoprotein E (*APOE*) are an important source of variability in metabolic disorders and influence the response to the pesticide chlorpyrifos (CPF). We aimed to study the epigenetic regulation on feeding control genes and the influence of postnatal CPF exposure, *APOE* genotype, and sex, and how these modifications impact on the metabolic response to a high-fat diet (HFD). Both male and female apoE3- and apoE4-TR mice were exposed to CPF on postnatal days 10–15. The DNA methylation pattern of proopiomelanocortin, neuropeptide Y, leptin receptor, and insulin-like growth factor 2 was studied in the hypothalamus. At adulthood, the mice were given a HFD for eight weeks. The results highlight the importance of sex in the epigenetic regulation and the implication of CPF treatment and *APOE* genotype. The body weight progression exhibited sex-dimorphic differences, apoE4-TR males being the most susceptible to the effects induced by CPF and HFD. Overall, these results underscore the pivotal role of sex, *APOE* genotype, and developmental exposure to CPF on subsequent metabolic disturbances later in life and show that sex is a key variable in epigenetic regulation.

## 1. Introduction

Obesity has undeniably increased its worldwide prevalence in recent decades. It is a major risk factor for other common diseases such as type 2 diabetes, metabolic syndrome, and some types of cancer [1]. According to the Developmental Origins of Health and Disease (DOHaD) hypothesis, early life factors such as diet, stress, or exposure to toxicants can increase vulnerability to chronic diseases later in life [2,3]. Hence, obesity can be modulated by the developmental alterations of metabolic programming and energy homeostasis [4]. Interestingly, the organophosphate pesticide chlorpyrifos (CPF), whose main mechanism of action is the inhibition of the enzyme acetylcholinesterase [5,6], can elicit neurotoxic effects and modulate metabolic homeostasis. CPF is a worldwide used pesticide for crop protection and the general population is mainly exposed to it through their diet [7]. In previous studies in rodents, we found that animals fed a CPF-supplemented diet during adulthood presented a phenotype resembling obesity in a genotype-dependent manner [8,9]. More specifically, CPF-treated mice presented increased glucose and insulin levels in plasma, higher insulin resistance, and greater ghrelin levels than their control peers [8]. In recent in vitro investigations, we also found that CPF can enhance adipocyte differentiation in a culture of undifferentiated murine pre-adipocytes and increase their ability to store lipids [10].

Remarkably, the human polymorphic apolipoprotein E (*APOE*) gene, which encodes for a protein involved in lipid transport, presents three main isoforms in humans: apoE2, apoE3, and apoE4 [11]. These isoforms are an important source of variability in responses to toxic insults and diet variations. *APOE* genotypes have different metabolic profiles, with different susceptibilities to obesity and insulin resistance. For instance, mice expressing the human form of *APOE3* presented a higher body weight than their *APOE4* counterparts after a high-fat diet [12,13,14], which can be attributed to a more efficient use of nutrients by animals expressing the apoE3 isoform [13]. In turn, a higher risk of developing impaired tolerance to glucose and diabetes mellitus-like metabolic traits was observed in *ε4* carriers [12,14]. The different isoforms of the *APOE* gene also have different responses to toxic insults, including exposure to CPF [15,16]. The greater vulnerability of mice carrying the human form of the *APOE3* gene to developing an obese-like phenotype [8,9] is explained by the capacity of CPF to disrupt the homeostasis of insulin and leptin signaling pathways, so the incidence in this genotype is greater [17].

Early life exposure to CPF could predispose to the development of metabolic alterations later in life [18]. Developmental exposure to CPF has shown long-term effects over a broad spectrum of functions, including learning and memory [16,19], as well as the cerebral expression of cholinergic elements [16,20]. Since the long-term effects of developmental exposure are regularly observed, epigenetic regulation—including DNA methylation, histone modifications and micro-RNA activity—has emerged as a plausible mechanism. In fact, DNA methylation is the most studied epigenetic mechanism and consists of the addition of a methyl group to a cytosine within a CpG dinucleotide in the DNA sequence [21]. The methylation of specific zones, generally in the promoter region, can modulate transcription and regulate gene expression [22].

While cognitive and motor deficits caused by CPF have been the object of considerable study, less attention has been paid to the link between this pesticide and metabolic disturbances. Feeding behavior, body weight (BW), and energy expenditure are primarily regulated by the hypothalamus, in close communication with peripheral and central signals. The neurons in the arcuate nucleus (ARC) are important responders to these signals [23]. In rodents, neurons in the ARC proliferate and differentiate during the embryonic period and send axons to their targets during the postnatal weeks prior to weaning [24,25]. These early developmental periods are especially sensitive to environmental factors and program metabolic tone in adults [26]. Two different types of neurons, mainly found in the ARC of the hypothalamus synthesize either anorexigenic or orexigenic neuropeptides in response to leptin, ghrelin, and insulin signaling [27,28,29]. Leptin, a hormone secreted in the adipose tissue, binds to leptin receptors (LEPR) in the hypothalamus and triggers multiple signaling cascades. This stimulates the synthesis of anorexigenic neuropeptides—proopiomelanocortin (POMC) and cocaine-and-amphetamine regulated transcript (CART)—and inhibits the synthesis of orexigenic neuropeptide Y (NPY) and agouti-related peptide (AgRP) [27,30]. Ghrelin, an orexigenic peptide hormone, can also regulate appetite by binding to the ghrelin receptor (GHSR1α) in the hypothalamus, which promotes food intake and adiposity [31]. Furthermore, insulin-like growth factor 2 (IGF2), an imprinted gene mostly known for its implication in development, can play a role in the hypothalamic regulation of appetite by inhibiting the orexigenic action of NPY [32].

Taking the above into account, to examine long-term altered energy homeostasis induced by CPF, in this study we investigated DNA methylation as the mechanism of epigenetic regulation of several genes in the hypothalamic area, which is involved in feeding control and energy balance. The study was conducted in a transgenic mouse model for the human *APOE3* and *APOE4* genotype exposed to CPF during the postnatal period. Thereafter, we introduced a high-fat diet to focus on how these factors influenced metabolic homeostasis in an obesogenic environment.

## 2. Material and Methods

### 2.1. Animals and Care

**Figure 1:**
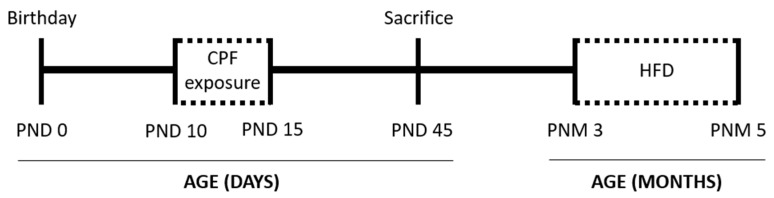
Experimental timeline including the treatment period from postnatal days (PND) 10 to 15 and sacrifice to obtain biological samples at PND 45. At postnatal month (PNM) 3, the animals were subjected to the high-fat diet (HFD) until PNM 5. Abbreviations: CPF: chlorpyrifos.

Male and female apoE-TR mice, homozygous for the *ε3* and *ε4* alleles, respectively (Taconic Europe, Lille Skensved, Denmark), were used. This apoE-TR animal model has a C57BL/6 background, with the human allele apoE replacing the murine gene [33]. The animals were housed under a 12 h light/dark automatic light cycle (light on 8:00–20:00) in standard conditions (temperature of 22 ± 2 °C and humidity of 50 ± 10%), with 2–5 individuals of the same genotype per cage. All mice were allowed free access to fresh water and were fed a normal chow diet (Panlab, Barcelona, Spain). The experimental timeline is shown in Figure 1. The use of animals and the experimental protocol were approved by the Animal Care and Use Committee of the Rovira i Virgili University (Tarragona, Spain). The experiments were conducted in accordance with Spanish Royal Decree 53/2013 on the protection of experimental animals, and the European Communities Council Directive (2010/63/EU).

### 2.2. Chemicals and Treatment

CPF [0,0-diethyl O-(3,5,6-trichloropyridin-2-yl) phosphorothioate], purity 99.5%, was obtained from Sigma-Aldrich Co. LLC (Madrid, Spain). The compound was dissolved in corn oil and adjusted in order to administer 1 mg/kg in 1 μL/g of body weight. We distributed the animals in two different groups: the CPF-treated group was orally treated with CPF on postnatal days (PND) 10–15 using a micropipette, while the control group was given the vehicle for the same period. The animals were periodically monitored and maintained under standard conditions.

### 2.3. Brain Sampling

At PND 45, biological samples were collected. The animals were deeply anesthetized with isoflurane using a SomnoSuite Low Flow Vaporizer anesthesia system (Kent Scientific, Torrington, CT, USA) and then euthanized by decapitation. Whole brains were collected and dissected to obtain the hypothalamus. Samples were snap frozen in liquid nitrogen and immediately stored at −80 °C until analysis.

### 2.4. Isolation of DNA and RNA

DNA and RNA were simultaneously extracted from the hypothalamus samples, using an Allprep DNA/RNA Mini Kit (Qiagen Inc., Hilden, Germany). The nucleic acid concentration and purity were measured using a Nanodrop 2000c spectrophotometer (ThermoFisher Scientific, Waltham, MA, USA). We included the following groups: control apoE3-TR males (n = 5), control apoE3-TR females (n = 6), CPF-treated apoE3-TR males (n = 6), CPF-treated apoE3-TR females (n = 6), control apoE4-TR males (n = 6), control apoE4-TR females (n = 6), CPF-treated apoE4-TR males (n = 6) and CPF-treated apoE4-TR females (n = 6).

### 2.5. Methylation Analysis: Bisulfite Modification and Pyrosequencing

A total of 500 ng of genomic DNA was used for bisulfite conversion using an EZ DNA Methylation-Gold Kit (Zymo Research, Irvine, CA, USA). Bisulfite sequencing primers for the different genes were designed using PyroMark assay design software (Qiagen Inc., Hilden, Germany). Because of its influence on the transcription of the gene, the assays prioritized the study of the promoter region. The details for the primer sequences are summarized in Table 1. PCRs were carried out for each gene in a total volume of 25µL. Methylation was determined by pyrosequencing on a PyroMark Q48 (Qiagen Inc., Hilden, Germany). The results were analyzed by PyroMark Q48 Autoprep 2.4.2. Software (Qiagen Inc., Hilden, Germany).

### 2.6. Gene Expression Analysis

The gene expression of *Pomc*, *Npy*, *Lepr*, and *Igf2* was assessed by real-time polymerase chain reaction (qPCR). The complementary DNA (cDNA) was synthetized from 0.5 μg of RNA from each sample, using a Maxima First Strand cDNA Synthesis Kit for RT-qPCR (ThermoFisher Scientific, Waltham, USA). The qPCR for each gene was performed using a Maxima SYBR Green/ROX qPCR Master Mix (2X) kit (ThermoFisher Scientific, Waltham, USA) and Rotor-Gene Q Real-Time PCR cycler (Qiagen Inc., Hilden, Germany). The primers used are shown in Table 2. Rotor-Gene Q 2.0 software was used to calculate the cycle threshold (Ct) and the relative levels of expression of the target genes were normalized to housekeeping gene *Gapdh* with the 2^−ΔΔCt^ method.

### 2.7. Dietary Intervention: High-Fat Diet

At three months of age, another set of animals subjected to the same postnatal treatment, underwent a dietary intervention in order to better understand the extent of the epigenetic regulation of body weight and food intake in an obesogenic environment. Before the challenge, they were paired according to sex, genotype, and postnatal treatment. Body weight and food intake were measured twice a week for one week (week 0). The animals were then distributed in two dietary groups: the first one received a high-fat diet (HFD) (Purified diet 230 HF, SAFE, Augy, France). It contained 5.32 kcal/g, with 13.1% proteins, 26.3% carbohydrate and 60.6% fat. The second group was fed a standard maintenance chow diet (SAFE A04 diet, Panlab, Barcelona, Spain). This diet contained 3.34 kcal/g, with 19.3% proteins, 72.4% carbohydrates and 8.4% fat. The dietary intervention lasted for eight weeks, and body weight (g) and food intake were assessed twice a week. The number of animals in each group is shown in Table 3.

### 2.8. Statistical Analysis

Data were analyzed with the SPSS 26.0 software (IBM Corp, Chicago, IL, USA). The results of the methylation analysis and the gene expression were studied with a three-way analysis of variance (ANOVA), with sex, genotype, and treatment as the main factors. Pearson correlations were used to investigate associations between methylation and mRNA levels. The body weight was studied with an ANOVA for repeated measures. Tukey’s post-hoc test of variance was used to analyze differences between groups. The homogeneity of the variance was assessed by a Levene test. Statistical significance was set at *p* < 0.05. Results are reported as mean values ± S.E.M.

## 3. Results

### 3.1. DNA Methylation and Gene Expression

The methylation status was analyzed for each CpG position within the target region of the genes studied: *Pomc, Npy, Lepr,* and *Igf2*. Then, we assessed the mean methylation levels of the region studied by calculating the arithmetic mean of the analyzed CpG positions for each gene in order to get a better understanding of the methylation status. Eventually, gene expression was determined and the possible relation between methylation and gene expression was analyzed.

#### 3.1.1. *Pomc* Methylation is Highly Influenced by Sex while CPF Modulates Gene Expression Levels

**Figure 2:**
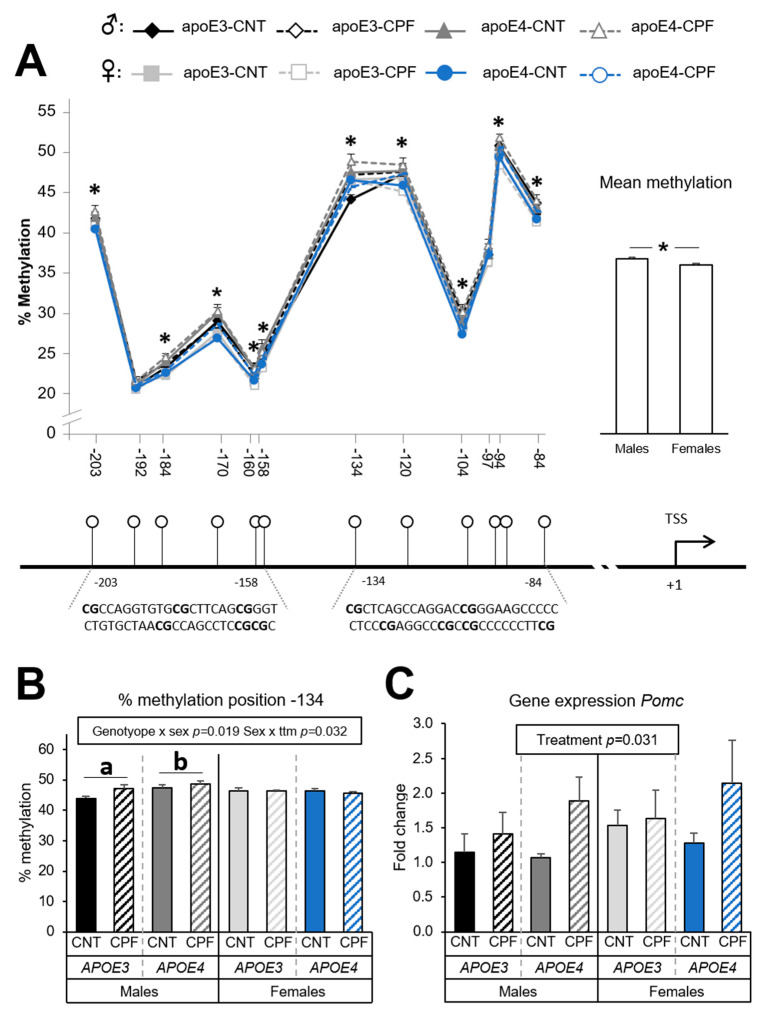
*Pomc* gene methylation at the CpG positions studied in the target zone in the promoter and overall methylation levels, which are sharing the same y axis. Below, there is the graphical representation of the CpG positions studied and the exact sequence of the target region (**A**). Methylation at the specific position −134 (**B**) and gene expression of the *Pomc* gene (**C**). All groups included 6 animals except for the *APOE3*-CNT that included 5. An asterisk (*) indicates significant differences between groups at *p* < 0.05. Abbreviations: CNT, control; CPF, chlorpyrifos-treated; ttm, treatment. Data is contained within the Supplementary File S1.

A total of 12 CpG positions were analyzed for the *Pomc* gene, all of them being in the promoter region. Differences in the percentage of methylation were assessed by a three-way ANOVA (sex × genotype × treatment) for each CpG site. Sex was observed to have a significant effect on methylation levels in positions −203 [F(1,39) = 6.086, *p* = 0.018], −184 [F(1,39) = 10.504, *p* = 0.002], −170 [F(1,39) = 19.466, *p* < 0.001], −160 [F(1,38) = 7.544, *p* = 0.009], −158 [F(1,39) = 5.100, *p* = 0.030], −120 [F(1,39) = 5.963, *p* = 0.019], −104 [F(1,39) = 4.947, *p* = 0.032], −94 [F(1,39) = 13.394, *p* = 0.001] and −84 [F(1,39) = 8.382, *p* = 0.006]. Methylation levels were highest for males. The study of the mean methylation of the 12 CpG positions in the *Pomc* promoter showed a significant sex difference [F(1,37) = 7.572, *p* = 0.009], with males presenting higher methylation levels than females (Figure 2A). An interaction between genotype and sex [F(1,39) = 5.978, *p* = 0.019] and between sex and treatment [F(1,39) = 4.931, *p* = 0.032] was observed in CpG position −134. Further analysis by a one-way ANOVA (genotype) for each sex revealed that apoE4-TR males exhibited a higher percentage of methylation than apoE3-TR males (Figure 2B). Interestingly, apoE3-TR males were more susceptible to the effects of CPF, having higher methylation levels than their control counterparts. This effect was not observed in females. On the other hand, the gene expression results revealed that the treatment had a significant effect [F(1,38) = 4.991, *p* = 0.031], with the CPF-treated groups presenting higher mRNA expression levels than control (Figure 2C). The Pearson correlation test showed no significant correlations between methylation levels and the gene expression results.

#### 3.1.2. CPF Treatment Modulates *Npy* Methylation Only in Males

**Figure 3:**
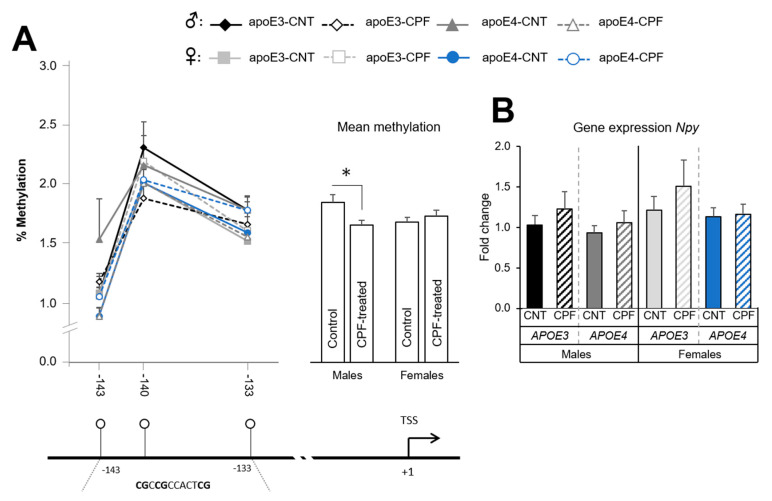
*Npy* gene methylation at the CpG positions studied in the target zone in the promoter and overall methylation levels, which are sharing the same y axis. Below, there is the graphical representation of the CpG positions studied and the exact sequence of the target region (**A**). Gene expression of the *Npy* gene (**B**). All groups included 6 animals except for the *APOE3*-CNT that included 5. An asterisk (*) indicates significant differences between groups at *p* < 0.05. Abbreviations: CNT, control; CPF, chlorpyrifos-treated. Data is contained within the Supplementary File S1.

The assay designed for *Npy* covered a total of seven CpG positions, all in the promoter region. However, we could only trust three of them because of technical issues with the remaining four positions. The study of each position found no differences between groups, although the mean methylation of the region revealed significant differences. More specifically, we observed an interaction between sex and postnatal treatment [F(1,37) = 5.489, *p* = 0.025], with CPF-treated males exhibiting lower methylation than control males (Figure 3A). The results of the gene expression analysis showed no significant differences between groups (Figure 3B). Nevertheless, a negative correlation was found between the mean methylation and mRNA levels in males (r = −0.466, *p* = 0.029), while a positive correlation was found in females (r = 0.740, *p* < 0.001).

#### 3.1.3. *Lepr* Presents Sexual-Dimorphic Differences only in the *APOE3* Genotype

**Figure 4:**
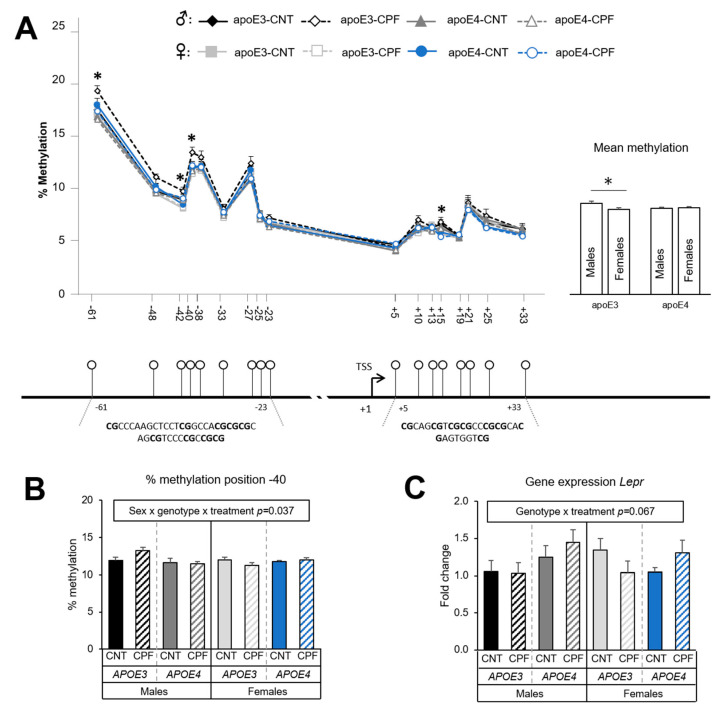
*Lepr* gene methylation at the CpG positions studied in the target zone in the promoter and overall methylation levels, which are sharing the same y axis. Below, there is the graphical representation of the CpG positions studied and the exact sequence of the target region (**A**). Methylation at the specific position −40 (**B**) and gene expression of the *Lepr* gene (**C**). All groups included 6 animals except for the *APOE3*-CNT that included 5. An asterisk (*) indicates significant differences between groups at *p* < 0.05. Abbreviations: CNT, control; CPF, chlorpyrifos-treated. Data is contained within the Supplementary File S1.

A total of 17 CpG positions were analyzed in the case of *Lepr*, distributed within the promoter. Differences in the percentage of methylation were assessed by a three-way ANOVA (sex × genotype × treatment) for each CpG site. Sex was found to have a significant effect at positions −42 [F(1,39) = 7.888, *p* = 0.008] and +15 [F(1,39) = 11.080, *p* = 0.002], with males showing higher methylation levels. An interaction between genotype and sex was observed at position −61 [F(1,39) = 7.809, *p* = 0.008], position −42 [F(1,39) = 4.181, *p* = 0.048], and position −40 [F(1,39) = 4.902, *p* = 0.033]. The study of the mean methylation levels of the 17 CpG positions in the *Lepr* promoter showed a tendency towards an interaction between sex and genotype [F(1,39) = 3.844, *p* = 0.057], which suggests a higher percentage of methylation in apoE3-TR males than in apoE3-TR females [F(1,23) = 5.698, *p* = 0.028] (Figure 4A). Moreover, CpG site −40 showed a triple interaction between sex, genotype and treatment [F(1,39) = 4.672, *p* = 0.037] as well as a tendency towards higher methylation levels in CPF-treated apoE3-TR males than in their respective control group (Figure 4B). On the other hand, gene expression results revealed a tendency towards an interaction between genotype and treatment [F(1,38) = 3.543, *p* = 0.067]. Significant differences were found between apoE3- and apoE4-TR mice only in those animals that had been treated with CPF [F(1,18) = 4.603, *p* = 0.046]. The CPF-treated apoE4-TR group presented a higher expression than mice in the CPF-treated apoE3-TR group (Figure 4C). The Pearson correlation test showed a negative correlation between the mean methylation of the target region and mRNA levels (r = −0.331, *p* = 0.026). Moreover, a negative correlation of CpG positions −40 (r = −0.345, *p=* 0.020) and −61 (r = −0.329, *p* = 0.027) with the mRNA levels showed the influence of these two positions on the transcription of the *Lepr* gene.

#### 3.1.4. *Igf2* Methylation Levels Depend on Sex

**Figure 5:**
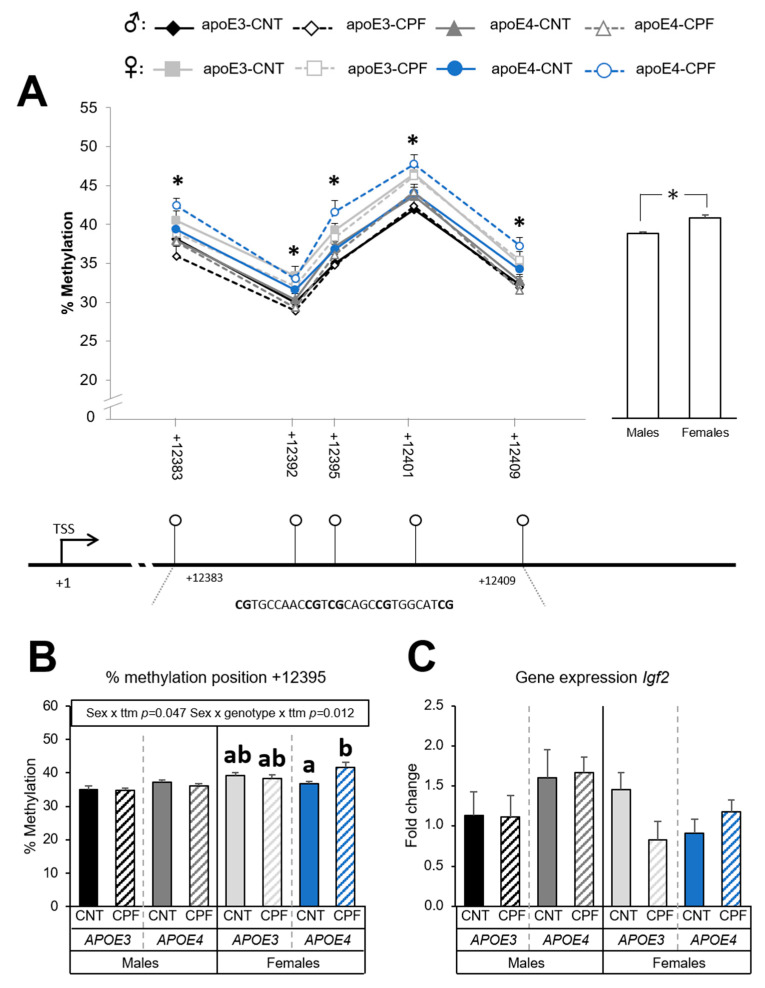
*Igf2* gene methylation at the CpG positions studied in the target zone in the DMR2 and overall methylation levels, which are sharing the same y axis. Below, there is the graphical representation of the CpG positions studied and the exact sequence of the target region (**A**). Methylation at the specific position +12395 (**B**) and gene expression of the *Igf2* gene (**C**). All groups included 6 animals except for the *APOE3*-CNT that included 5. An asterisk (*) indicates significant differences between groups at *p* < 0.05. Different letters (a, b) represent significant differences at *p* < 0.05. Abbreviations: CNT, control; CPF, chlorpyrifos-treated; ttm, treatment. Data is contained within the Supplementary File S1.

Five CpG positions were analyzed in the *Igf2* gene, all of which were in the DMR2 region. DMR2 is a well-studied intragenic regulatory region that is highly sensitive to methylation [40]. The differences in the percentage of methylation were assessed by a three-way ANOVA (sex × genotype × treatment) for each CpG site. Sex was noted to have a significant effect on methylation levels at all the positions: +12383 [F(1,39) = 14.485, *p* < 0.001], +12392 [F(1,39) = 17.818, *p* < 0.001], +12395 [F(1,39) = 26.425, *p* < 0.001], +12401 [F(1,39) = 14.518, *p* < 0.001] and +12409 [F(1,39) = 20.440, *p* < 0.001]. Levels were higher in females. Likewise, the study of the mean methylation levels in the five CpG positions in the DMR2 region of *Igf2* gene showed significant sex differences [F(1,39) = 27.958, *p* < 0.001], with females showing higher methylation percentages than males (Figure 5A). Interactions between sex and treatment [F(1,39) = 4.191, *p* = 0.047] and between sex, genotype and treatment [F(1,39) = 7.018, *p* = 0.012] were observed at CpG position +12395. Further analysis with a one-way ANOVA (group) followed by a post-hoc analysis for each sex found that expression was higher in the CPF-treated apoE4-TR females (Figure 5B). The results of the *Igf2* gene expression showed no significant differences between groups (Figure 5C), while no significant correlations were found between methylation and mRNA results.

### 3.2. Dietary Challenge: High-Fat Diet

#### Body Weight Progression is Influenced by CPF Exposure Only for the *APOE4* Genotype

**Figure 6:**
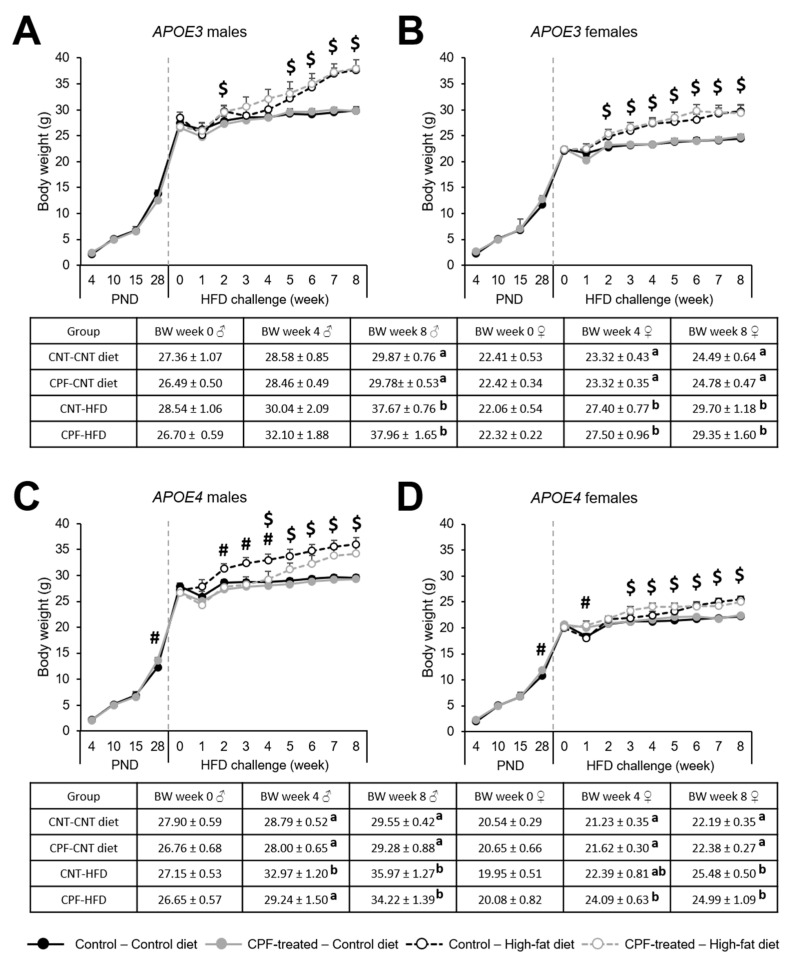
Body weight progression during the postnatal period and the high-fat diet for *APOE3* males (**A**), *APOE3* females (**B**), *APOE4* males (**C**), and *APOE4* females (**D**), with the BW of week 0, week 4 and week 8 described in the corresponding table. The symbol $ indicates a significant effect of the diet at *p* < 0.05, while the symbol # represents a significant effect of the treatment at *p* < 0.05. Different letters (a, b) represent significant differences at *p* < 0.05. Abbreviations: CNT, control; CPF, chlorpyrifos-treated; HFD, high-fat diet. Data is contained within the Supplementary File S1.

Body weight progression during the postnatal period (PND 4 to PND 28) was assessed by a three-way ANOVA (sex × genotype × treatment) for repeated measures, with time as the within-subject factor. We observed a significant effect of time [F(3,87) = 2477.340, *p* < 0.001], an interaction between time and sex [F(3,87) = 10.081, *p* < 0.001], between time, genotype, and treatment [F(3,87) = 3.223, *p* = 0.026], and between time, sex, genotype, and treatment [F(3,87) = 3.843, *p* = 0.012]. For this reason, from this point on, we studied the differences for each genotype and sex separately. A one-way ANOVA (treatment) revealed treatment differences at PND 28 in apoE4-TR mice (males: [F(1,29) = 13.525, *p* = 0.050]; females: [F(1,30) = 5.861, *p* = 0.022]). Animals in the CPF-treated group presented significantly higher body weights than those in control group (Figure 6C,D).

Body weight progression during the 8-week HFD challenge was studied by a two-way ANOVA (treatment × diet) for repeated measures, with time as the within-subject factor. We found a significant effect of time (*p* < 0.050) and a significant interaction between time and diet (*p* < 0.050) in all groups. In order to further study the weekly development of BW, a two-way ANOVA (treatment × diet) was used to assess the weekly differences between groups. ApoE3-TR males presented a permanent effect of the diet from week 5 on, whereas in apoE3-TR females this effect was observed from week 2 onwards (Figure 6A,B). In apoE4-TR males a significant effect of the CPF treatment was observed during the first weeks of the HFD (week 2 to week 4), when CPF-treated animals fed a HFD did not increase their BW in the same way as their control pairs. However, the influence of the diet was observed from week 4 until the end of the challenge (Figure 6C). The diet affected apoE4-TR females between week 3 and week 8 (Figure 6D). These results showed that CPF exposure had an impact only on apoE4-TR males.

## 4. Discussion

The present investigation was designed to study how *APOE* genotype, sex, and postnatal exposure to the pesticide CPF regulates the methylation of genes involved in feeding control, and how these epigenetic modifications can condition the subject’s outcomes in an obesogenic environment with a HFD. In recent studies, we have reported the capacity of CPF to alter insulin and leptin signaling pathways [17], and to increase body weight in a genotype-dependent manner [8,9]. Even though the underlying mechanisms have not been yet completely elucidated, we hypothesize that epigenetic modifications may be involved. Previous studies have demonstrated that the genes integrating the feeding control pathway in the hypothalamus can be highly influenced by epigenetic regulations [23]. Furthermore, epigenetic modifications are especially relevant during critical developmental periods and can impact long-term health [41,42,43]. To the best of our knowledge, the current study has been the first one assessing how CPF epigenetically regulates genes involved in the feeding control pathway and the subsequent life-long metabolic implications, in a sex- and *APOE*-dependent manner.

Environmental factors can influence the methylation pattern of genes during critical developmental periods. It has been reported that the appetite-regulating neuropeptides *Pomc* and *Npy* have been epigenetically modified by dietary interventions such as postnatal overfeeding [44], high-fat diet feeding [45,46,47,48], or dietary restrictions [49]. Moreover, gestational exposure to environmental pollutants such as the synthetic antibacterial triclosan altered *Pomc* promoter methylation and gene expression in mice, linked to a postnatal hyperphagic obesity [50]. In the same line, genome-wide DNA methylation studies in humans have found a link between higher exposure to organophosphate pesticides and modifications in the methylation of several genes [51,52]. Nevertheless, the specific modifications in methylation induced by CPF in the genes involved in energy homeostasis remain unascertained.

For the study of the methylation pattern of the gene coding for the anorexigenic neuropeptide POMC, we targeted two consecutive regions in the promoter analogous to those investigated in previous studies on mice [49,53,54]. Significant differences between groups were found at ten CpG positions, most of which were highly influenced by sex. In fact, males showed a higher methylation percentage than females. This is in agreement with the premise that the regulation of several aspects of energy homeostasis depends on the sex [55]. Some positions can be in the binding site for specific transcription factors, so they may have more influence on regulating gene expression. This is the case of specificity protein 1 (Sp1), whose binding site includes CpG position −94 in the promoter of the *Pomc* gene. Sp1 is critical for leptin signal transduction after leptin binds to *Lepr*: Sp1 binds to *Pomc* and interacts with phosphorylated STAT3 to regulate *Pomc* promoter activity [56]. Therefore, hypermethylation at this position interferes with the Sp1 binding and decreases *Pomc* gene expression [54]. Previous studies in rats have found that this position is a target for methylation modifications in response to dietary interventions [44,46]. In the current investigation, we found that methylation in this specific position was higher in males than in females. Despite the fact that sex differences have not been extensively studied, with most of the studies focusing on one sex, a recent study including both males and females found subtle sex differences in the *Pomc* methylation pattern [48].

On the other hand, the current study found higher levels of *Pomc* gene expression in the CPF-treated group but no significant correlations between the mRNA and methylation levels. Even though some studies have found a negative correlation between methylation status and gene expression [57,58], others, like ours, have not [46,53,59]. A plausible explanation for this discrepancy could be the involvement of other epigenetic mechanisms or the influence of other regions in the regulation of *Pomc* gene expression. It is also worth noting that *Pomc* is under the regulatory control of leptin [30], so we hypothesize that changes in the leptin levels are necessary to observe significant effects on *Pomc* expression.

We then studied the epigenetic regulation of the gene coding for the orexigenic neuropeptide NPY. The *Npy* gene promoter presented low methylation levels, up to 2.5%, which is in accordance with the results of previous studies [44]. Despite these low levels and the fact that only three CpG positions were analyzed, we observed that the mean methylation levels are influenced by sex and treatment. More specifically, CPF-treated males presented lower methylation levels than their respective controls, suggesting that males are more sensitive than females to CPF. This sexual-dimorphic sensitivity has been also observed with other toxics such as the pesticide tributyltin [60]. Along the same lines, the male but not female offspring of dams exposed to ambient fine particles (PM_2.5_) showed higher expression of the hypothalamic *Npy* gene [61]. Higher expression of *Npy* in CPF-treated males would lead to an upregulation to higher food intake and BW, which is in agreement with previous observations of CPF inducing increased BW in adult mice [8,9].

The methylation analysis of *Lepr* showed sexual dimorphic differences in the *APOE3* genotype, with higher mean methylation levels in males. In addition, CpG position −40 also presented significant differences between males and females, being methylation levels of apoE3-TR males higher than those of apoE4-TR males. Altogether, these observations highlight the influence of the *APOE* genotype, and its interaction with sex, on the *Lepr* signaling pathway in the hypothalamus. Interestingly, the apoE gene has been reported to play a role in the regulation of food intake [62,63]. Firstly, apoE is highly expressed in the ARC and PVN of the hypothalamus [62]. Secondly, the intraventricular injection of apoE decreased food intake and BW in rats, whereas the injection of an apoE antiserum increased these same parameters [62]. Thirdly, a link has been reported between apoE levels and leptin signaling throughout the *Lepr* in ARC astrocytes [63]. Considering all the evidence, it is not surprising that the *APOE* isoform can lead to differences in the leptin signaling pathway.

Gene expression analysis of *Lepr* in the hypothalamus has found a tendency towards an interaction between genotype and treatment, which highlights the potential influence of CPF on leptin signaling. Previous studies with males have reported that the *APOE* genotype determines the response to CPF in terms of the hepatic protein expression of the SOCS3 and the ratio between phospho-STATS3 and total STATS3, both involved in the leptin signaling pathway [17]. Indeed, CPF-treated apoE3-TR males presented higher levels than apoE4-TR males [17]. Interestingly, in the present investigation the results of gene expression negatively correlated with the data of methylation analysis, suggesting that higher methylation inhibits gene expression and vice-versa. These findings support the implication of epigenetic modification in the regulation of *Lepr* gene expression in the hypothalamus.

In the case of the *Igf2* gene the region selected to study the methylation pattern was the DMR2. The results showed a clear effect of sex, with females presenting higher methylation levels than males. Gene expression analysis showed no significant differences between groups nor a correlation with methylation results. The study of *Igf2* methylation levels in several tissues also showed sex- and age-dependent differences between groups [64] and the higher methylation of *Igf2* has been associated with lower BMI [65]. Indeed, *Igf2* is mainly known for its role in embryonic development. However, it has also been reported that it contributes to the control of energy homeostasis. In fact, *Igf2* and its receptors are expressed in the hypothalamus, more specifically in the PVN [66]. Intracerebroventricular infusions of *Igf2* in rats showed a dose-dependent decrease in food intake and BW [67] by decreasing the release of *Npy* in the hypothalamus in a similar way to insulin [32].

Overall, the results of the epigenetic and gene expression analysis revealed that sex has a considerable influence on the methylation pattern of the genes studied and, to a lesser extent, so do *APOE* genotype and postnatal exposure to CPF. These results highlight the importance of the sexual-dimorphic regulation of the hypothalamus circuits involved in metabolic homeostasis. Increasing evidence indicates important differences between males and females in the hypothalamic control of energy metabolism [55,68,69] and different sensitivities to environmental factors [61]. Various studies in humans have also reported an overall influence of sex on methylation status [70,71,72], supporting the importance of the current results.

In the scientific literature, there is extensive evidence on how dietary interventions can alter epigenetic regulation. However, there is less information about how existing modifications can condition the response to an environmental challenge. We hypothesized that the differences in methylation above discussed could condition the individual response to an obesogenic environment such as the introduction of a high-fat diet. Certainly, the introduction of a HFD over a long period of time would lead to metabolic changes, but the eventual response depends on individual features [73]. In the present study, we found that both male and female CPF-treated apoE4-TR mice showed significantly higher BW than their respective controls at PND 28. This effect was not noticed in the apoE3-TR mice that had been subjected to the same postnatal treatment. An *APOE* genotype specific effect of CPF treatment was also observed by Peris-Sampedro et al. (2020) [74] at the exact same period in apoE4-TR but not in C57BL/6 females. These observations suggest a greater vulnerability of apoE4-TR mice to BW alterations at young ages.

During the dietary intervention, most of the groups presented a time- and diet- dependent increase in BW. However, CPF-treated apoE4-TR males presented a different response to the diet for the first three weeks, suggesting that subtle basal differences intrinsic to the *APOE4* genotype could be exacerbated by a dietary intervention. In a previous investigation, we found that a scheduled restricted presentation of a high-fat diet induced a higher intake of palatable food in female mice postnatally exposed to CPF, being apoE4-TR mice the most sensitive to the discontinuation of the diet, with a higher “wanting” response with refeeding [74]. Therefore, the fact that we did not observe a similar pattern in the current study suggests that a scheduled restricted presentation of a high-fat diet may have subtle effects that cannot be observed with unrestricted access to the diet, which highlights the importance of the administration protocol. The introduction of a HFD can increase leptin levels in male and female rats [75], and also increases BW and insulin in mice [13]. In turn, Lassiter and Brimijoin (2008) [76] found increased BW in male but not female adult rats exposed to CPF during gestation, while studies in rats developmentally exposed to a different organophosphate pesticide, parathion, found that a HFD aggravates the effects of the pesticide on metabolic parameters [77]. Therefore, we can infer that the synergic effect of the *APOE4* genotype and developmental exposure to CPF might predispose apoE4-TR males to a different metabolic response to the introduction of an HFD.

Lassiter and Brimijoin (2008) [76] observed a loss of correlation between BW and leptin levels in serum after CPF exposure, and put forward the leptin pathway as a potential mechanism underlying this loss. Interestingly, the leptin surge in rodents peaks on PND 9—PND 10 [78]. It has been reported that modifications in this period may induce long-term effects on leptin signaling [79]. In this sense, Attig et al. (2008) [80] found that disrupting leptin during the first two postnatal weeks in female rats induced leptin resistance during adulthood, and predisposed them to diet-induced obesity. Therefore, we can hypothesize that the CPF-triggered disruption of leptin levels during the postnatal period may contribute to the effects of treatment observed later in life. In parallel, *Lepr* mRNA has been observed to be higher in young apoE4-TR than in apoE3-TR mice [81]. As mentioned above, the *APOE4* genotype and CPF influence the gene expression levels of *Lepr* in the hypothalamus, which negatively correlated to the methylation levels in the *Lepr* promoter. Finally, this dysregulation in apoE4-TR mice can be also linked to the differences observed in *Npy* methylation levels after treatment on males, which may be an overcompensation caused by a CPF-triggered alteration in the leptin pathway.

## 5. Conclusions

To sum up, epigenetic modifications and gene expression are modulated by several factors, which lead to different long-term regulations. Sex plays an unequivocally important role in the methylation pattern of genes involved in appetite regulation. Its influence may underlie the sex-dependent differences in basal BW. In fact, males and females presented a different BW progression pattern during the dietary challenge. Remarkably, the *APOE* genotype was an important factor in the epigenetic regulation of *Lepr* and its contribution to possible metabolic alterations deserves further investigations. With respect to the progression of BW, apoE4-TR males presented higher susceptibility to the cumulative effects of both HFD and postnatal CPF treatment. Moreover, apoE4-TR male mice also showed differences in the methylation of *Npy* after CPF exposure. In conclusion, developmental exposure to CPF, together with individual differences involving sex and the *APOE* genotype, may contribute to the global incidence of overweight and obesity.

## Figures and Tables

**Table 1:** Sequence of primers used in the methylation analysis.

Gene	Sequences 5′–3′
*LepR*	F: Biotin-GTTGTAAAGGTTAGAGAGGATAGAAT
	R: AACTAAAAAAATATCCCACCTATATCC
	Seq1: ACACTAACCACTCAAAT
	Seq2: ACTCTATACTACTAACTCAAAA
*Pomc*	F: Biotin-TAGGGTTGGGTGGGTGAG
	R: CACAAAAACCCTAAACCTCTATCCAATTCT
	Seq1: CTTTCCAAACAAATATACCTT
	Seq2: ATTAAATTCTTCCTAACCAC
*Igf2 DMR2*	F: GGGTTGGGGTGGTTATTTTAATGG
	R: gacgggacaccgctgatcgtttaATCTCTTTATCTCACCCCATAATTC
	Universal biotinylated primer: Biotin-gggacaccgctgatcgttta
	Seq1: GAATTTTTAGGTAGGTTTTTAAG
*Npy*	F: GGGTAGTTTAGAATTGGGGTGTGG
	R: ATAACTCCCACAACCACTTC-Biotin
	Seq1: AATTGGGGTGTGGGT

Primer sequences are of our own design, except for the *Igf2 DMR2,* which was designed by Freitag et al. (2014) [34].

**Table 2:** Sequence of primers used in the gene expression analysis.

Gene	Forward Sequence	Reverse Sequence	Reference
*LepR*	CGTGGTGAAGCATCGTACTG	GGGCCATGAGAAGGTAAGGT	[35]
*Pomc*	AGAACGCCATCATCAAGAAC	AAGAGGCTAGAGGTCATCAG	[36]
Npy *	TGGACTGACCCTCGCTCTAT	GTGTCTCAGGGCTGGATCTC	[37]
*Igf2*	ACACGCTTCAGTTTGTCTGTTC	GGGGGTGGCACAGTATGTC	[38]
*Gapdh*	ACAACTTTGGCATTGTGGAA	GATGCAGGGATGATGTTCTG	[39]

Primer sequences have been evaluated by Primer BLAST. Genes with an asterisk (*) are described for another type of rodent. These sequences were checked and matched to the mouse genome.

**Table 3:** Number of animals in each group in the high-fat diet (HFD) challenge.

Genotype	Treatment	Males	Females
*APOE3*	CNT—CNT	8	8
	CNT—HFD	8	8
	CPF—CNT	8	8
	CPF—HFD	8	8
*APOE4*	CNT—CNT	8	8
	CNT—HFD	8	8
	CPF—CNT	12	8
	CPF—HFD	7	10

Abbreviations: CNT, control; CPF, chlorpyrifos-treated; HFD, high-fat diet.

## Data Availability

The data presented in this study are available in the Supplementary Material.

## Supplementary Materials

The following are available online at https://www.mdpi.com/1660-4601/18/1/184/s1, File S1: Research data.
